# Profiling (placental) DNA methylation in cell-free DNA across gestation: the Rotterdam Periconception Cohort

**DOI:** 10.1093/molehr/gaaf011

**Published:** 2025-05-08

**Authors:** Marjolein M van Vliet, Ruben G Boers, Joachim B Boers, Olivier J M Schäffers, Lotte E van der Meeren, Joost Gribnau, Sam Schoenmakers, Régine P M Steegers-Theunissen

**Affiliations:** Department of Obstetrics and Gynecology, Erasmus MC, Rotterdam, The Netherlands; Department of Developmental Biology, Erasmus MC, Rotterdam, The Netherlands; Department of Developmental Biology, Erasmus MC, Rotterdam, The Netherlands; Department of Developmental Biology, Erasmus MC, Rotterdam, The Netherlands; Department of Obstetrics and Gynecology, Erasmus MC, Rotterdam, The Netherlands; Department of Developmental Biology, Erasmus MC, Rotterdam, The Netherlands; Department of Pathology, Erasmus Medical Centre Rotterdam, Rotterdam, The Netherlands; Department of Pathology, Leiden University Medical Center, Leiden, The Netherlands; Department of Developmental Biology, Erasmus MC, Rotterdam, The Netherlands; Department of Obstetrics and Gynecology, Erasmus MC, Rotterdam, The Netherlands; Department of Obstetrics and Gynecology, Erasmus MC, Rotterdam, The Netherlands

**Keywords:** epigenetics, pregnancy, cell-free DNA, gestational age, DNA methylation, placenta

## Abstract

Placental DNA methylation varies across gestation and is associated with obstetrical complications. Cell-free DNA (cfDNA) from maternal plasma could provide a noninvasive approach to study placental DNA methylation in ongoing pregnancies. However, research on maternal cfDNA methylation is limited and technologically challenging. Therefore, we aimed to investigate DNA methylation in maternal cfDNA and placental tissues across gestation using the innovative methylation DNA sequencing (MeD-seq) technology. Secondly, we explored the origins of methylation differences in maternal cfDNA across gestation, and aimed to identify gestational age-associated placental DNA methylation markers directly in cfDNA. We longitudinally collected maternal cfDNA in all three trimesters and at birth (n = 10), alongside placental tissues from first trimester, second trimester, and term pregnancies (all n = 10), and used previously collected maternal blood buffy coat samples (n = 20). Different placental cell types, including syncytiotrophoblasts/cytotrophoblasts (SCTs/CTBs) (n = 10), extravillous trophoblasts (n = 7), and syncytial knotting (n = 3), and maternal cell types including spiral arteries (n = 3) and endometrial epithelium (n = 3), were isolated using laser capture microdissection. Differentially methylated regions (DMRs) identified in cfDNA from pregnant compared to non-pregnant women (n = 6) ranged from 798 to 2163 in first and third trimesters, respectively. Gradual DNA methylation changes were observed across gestation in cfDNA, placental tissues, and trophoblasts. We showed an increase in DMRs in cfDNA, that overlap with DNA methylation in placental tissues and especially trophoblasts, and in DNA methylation of placenta-specific markers across gestation, reflecting an increased placental-originated cfDNA fraction. Among 110 DMRs between first trimester and term placental tissues, those related to *NXPH4*, *EPS8L2*, *AMOTL1*, and *IRX2* had the strongest association with gestational age in cfDNA, for which comparable associations were found in SCTs/CTBs. These DMRs were all hypomethylated in maternal buffy coat samples. This study indicates the feasibility of identifying gestational age-dependent placental DNA methylation marks in maternal cfDNA and can serve as a reference for future studies.

## Introduction

DNA methylation is an epigenetic mechanism with a vital role in placental development and function (Nelissen *et al.*, 2011; Moore *et al.*, 2013; Januar *et al.*, 2015). Differences in placental DNA methylation profiles have been found in relation to gestational age (Novakovic *et al.*, 2011; Lee *et al.*, 2019; Yuan *et al.*, 2021; Zhang *et al.*, 2021) and have been associated with several obstetrical complications such as preeclampsia (Cirkovic *et al.*, 2020; Cruz de *et al.*, 2020). Hence, studying placental DNA methylation dynamics across pregnancy can increase our understanding of placental development in both health and disease and may lead to the development of clinically relevant biomarkers for associated complications.

Studies investigating placental DNA methylation at early- and mid-gestation are limited, since acquiring placental tissue during pregnancy is historically limited by both logistic and ethical barriers. Moreover, the need to obtain physical placental tissue limits the clinical applicability of studying placental DNA methylation during pregnancy. An alternative approach to investigate placental DNA methylation could be the analysis of cell-free DNA (cfDNA) in maternal plasma. Although most cfDNA fragments in maternal blood originate from maternal tissues, placental-originated cfDNA fragments can also be detected in maternal blood during pregnancy. Importantly, the epigenetic features of the placenta are preserved in placental-originated cfDNA fragments (Moss *et al.*, 2018; Lo *et al.*, 2021). Until now, cfDNA derived from maternal plasma has been already widely used for noninvasive prenatal testing (NIPT), an antenatal blood test primarily used to screen for fetal chromosomal abnormalities (Mackie *et al.*, 2017).

Studies investigating the maternal cfDNA methylome are limited and generally technologically challenging, due to degradation of DNA isolated from already limited amounts of cfDNA, as side-effect of the necessary bisulfite treatment (Li, 2021). In our previous study, we therefore used methylated DNA sequencing (MeD-seq). By using a methylation-dependent restriction enzyme, MeD-seq is well-compatible with low amounts of cfDNA while providing full genome-wide coverage (Boers *et al.*, 2018; Deger *et al.*, 2021; Bos *et al.*, 2023). MeD-seq has extensively been validated and compared to other techniques, including whole-genome bisulfite sequencing, MeDIP, and the 450K Infinium array (Boers *et al.*, 2018, 2023). We previously showed numerous methylation differences between cfDNA from non-pregnant women and pregnant women in the first trimester using MeD-seq (van Vliet *et al.*, 2025a). Other studies have reported differences in cfDNA methylation levels between trimesters (Jiang *et al.*, 2017), or estimated the placental-derived cfDNA fraction in different trimesters based on genome-wide cfDNA methylation profiles (Sun *et al.*, 2015; Del Vecchio *et al.*, 2021). In addition, a recent study showed the feasibility of predicting preeclampsia using the first-trimester maternal cfDNA methylome (De Borre *et al.*, 2023). However, genome-wide methylation dynamics in cfDNA in relation to the duration of gestation, and the possibility of detecting gestational age-associated placental methylation markers in cfDNA, have not been investigated before.

We hypothesized that both placental and maternal cfDNA methylation are altered with the duration of gestation and that placental DNA methylation differences related to gestational age can be detected in maternal cfDNA. Therefore, our first aim was to investigate DNA methylation in cfDNA from maternal blood and in placental tissues in association with gestational age using MeD-seq. Secondly, we aimed to explore the origins of methylation differences in maternal cfDNA across gestation, and to identify gestational age-associated placental DNA methylation markers directly in cfDNA. This study increases our understanding of the understudied cfDNA methylome across gestation and the role of epigenetic programming during normal (placental) development. Moreover, our data can serve as a reference for future studies including pregnancies with obstetrical complications such as preeclampsia.

## Materials and methods

### Ethics statement

All participants provided written informed consent, and our study was approved by the Medical Ethics Committee of the Erasmus MC (Predict study: MEC-2004-227, collection of first-/second-trimester placentas: MEC-2023-0028).

### Study design

We prospectively collected cfDNA from maternal plasma in all three trimesters and at birth and collected postpartum placental tissues from women who participated in the Rotterdam Periconceptional cohort (Predict study) (n = 10) at the Erasmus Medical Centre, Rotterdam (Steegers-Theunissen *et al.*, 2016; Rousian *et al.*, 2021; van Vliet *et al.*, 2025b). Women who were at least 18 years old with a singleton pregnancy and who were <10 + 0 weeks pregnant were eligible for participation. Blood samples were collected in each trimester, around 11, 22–25, and 30–33 weeks of gestation, and during delivery (n = 10). For the current study, blood samples were specifically selected from women who had an uncomplicated pregnancy (e.g. term pregnancy, birthweight percentile >10 and <90, no obstetrical complications such as hypertensive disorders of pregnancy, and no congenital malformations).

In addition to data collected within the Predict study, we prospectively collected placental tissues after elective abortions of singleton pregnancies without congenital abnormalities in a Dutch abortion clinic. These samples were collected from first- and second-trimester pregnancies at ∼9–12 weeks (n = 10) and 16–18 weeks (n = 10) of gestation. We further used our reference sets of cfDNA obtained from non-pregnant women (n = 6) and maternal buffy coat samples (leukocytes) (n = 20) as described previously ([dataset] van Vliet *et al.*, 2025a.

### Sample processing

Blood samples were collected in CellSave preservative tubes (Menarini Silicon Biosystems Inc, Huntingdon Valley, PA, USA). Plasma was isolated by two centrifugation steps (10 min at 1711–2000 *g* followed by 10 min at 12 000–16 000 *g*) within 96 h and stored at −80°C prior to cfDNA isolation (van Dessel *et al.*, 2017). Placental tissues were collected after surgical abortion (first and second trimester) and after term birth (Predict study) and stored in buffered 4% formaldehyde solution. Full-thickness biopsies were taken followed by embedding in formalin-fixed paraffin-embedded (FFPE) blocks. Histopathological examination of the placentas was performed by a perinatal pathologist (L.E.v.d.M.).

### Laser capture microdissection

To validate our bulk placental analyses and to evaluate the (placental) cell type of origin in the maternal cfDNA methylation signatures, we isolated specific placental cell types using laser capture microdissection (LCM). PALM membrane slides 1.0 PEN (ZEISS, Jena, Germany) were UV treated (254 nm) and coated with poly-L-lysine (0.1%) (Sigma-Aldrich, St Louis, MO, USA) according to the manufacturer’s protocol before use, to improve tissue adhesion to the membrane. Sections of 16 µm were cut from the FFPE blocks, mounted on the pretreated membrane slides, and incubated at 60°C for 2 h. Subsequently, the sections were dewaxed, dehydrated, and stained with hematoxylin. Regions of interest were annotated by a perinatal pathologist (L.E.v.d.M.) and included syncytiotrophoblasts and cytotrophoblasts (SCTs/CTBs) in first and second trimester and term placental tissues, extravillous trophoblasts (EVTs) in first- and second-trimester placental tissues, and syncytial knotting (SK) from term placental tissues. Furthermore, we isolated spiral arteries and endometrial epithelium as maternal tissues from first- and second-trimester placental tissues, to explore a possible contribution of these cell types to the maternal cfDNA signature in pregnancy. The annotated regions were cut using the Leica Laser Microdissection system and we dissected 5–20 mm^2^ per sample including a minimum of 1000 cells to obtain between 10 and 50 ng DNA per 8.66 µl of sample. DNA concentrations were measured using NanoDrop™.

### (cf)DNA isolation

cfDNA was isolated from 4 ml of plasma using the QIAamp Circulating Nucleic Acid Kit (QIAGEN, Hilden, Germany) according to the manufacturer’s protocol. For isolation of bulk placental DNA, four to six slides of 4–6 μm were cut from the FFPE blocks. One slide per sample was stained using hematoxylin and eosin, and DNA from normal placental parenchyma was isolated from subsequent slides. DNA isolation from both bulk placenta and LCM samples was performed using the QIAamp DSP DNA FFPE Tissue Kit (QIAGEN, Hilden, Germany) according to the manufacturer’s protocol.

### MeD-seq assay and data analysis

MeD-seq assays were performed as described previously (Boers *et al.*, 2018; Deger *et al.*, 2021; Bos *et al.*, 2023; van Vliet *et al.*, 2025a; van Vliet *et al*., 2025b). (cf)DNA was digested with the methylation-dependent restriction enzyme LpnPI (New England Biolabs, Ipswich, MA, USA) generating 32 bp DNA fragments containing the methylated CpG in the middle. LpnPI is unable to digest its own fragments preventing loss of fragments coming from CpG dense regions (Boers *et al.*, 2018). The TruPLEX DNAseq 96D kit (Rubicon Genomics, Takara Bio Europe, Saint-Germain-en-Laye, France) was used to prep samples for sequencing and samples were purified on a Pippin HT system with 3% agarose gel cassettes (Sage Science, Beverly, MA, USA). Samples were sequenced on the Illumina NextSeq2000 platform. Samples having <20 million reads or <20% CpG reads were considered failed. Dual indexed samples were demultiplexed using bcl2fastq software (Illumina, San Diego, CA, USA). Differentially methylated regions (DMRs) were identified using a previously established bioinformatics pipeline. Custom python scripts were used to process the acquired DNA methylation profiles. Raw fastq files were subjected to Illumina adaptor trimming. We filtered reads based on LpnPI restriction site occurrence between 13 and 17 bp from either the 5′ or 3′ end of the read to filter out DNA fragments that were methylated, and mapped reads that passed the filter to hg38 using bowtie2. BAM files were generated using SAMtools version 0.1.19 for visualization in IGV (Danecek *et al.*, 2021). To detect DMRs, a full genome-wide sliding window was used to detect sequentially differentially methylated LpnPI sites between two groups of the derivation cohort, and genome-wide read counts were normalized (RPM, reads per million) for coverage and compared using the chi-squared test. Neighboring significantly called LpnPI sites were binned and reported. Overlap of genome-wide detected DMRs was reported for TSS (1 kb before and 1 kb after), CpG island, or gene body regions (1 kb after TSS till TES) using the annotations of the UCSC database downloaded from ENSEMBL (Homo_sapiens_hg38.GRCh38.79.gtf, http://www.ensembl.org). A Bonferroni-corrected *P*-value ≤0.05 was considered statistically significant. *Z*-score transformation of the read count data was applied for unsupervised hierarchical clustering analyses.

To identify cfDNA methylation associated with duration of gestation, DMRs were identified between maternal cfDNA in each trimester and at delivery and cfDNA from non-pregnant women, as well as between trimesters and delivery in maternal cfDNA. Additionally, DMRs were identified between first trimester, second trimester, and term placental tissues and SCTs/CTBs, and between different trophoblast populations with a comparable gestational age.

**Figure 1.**
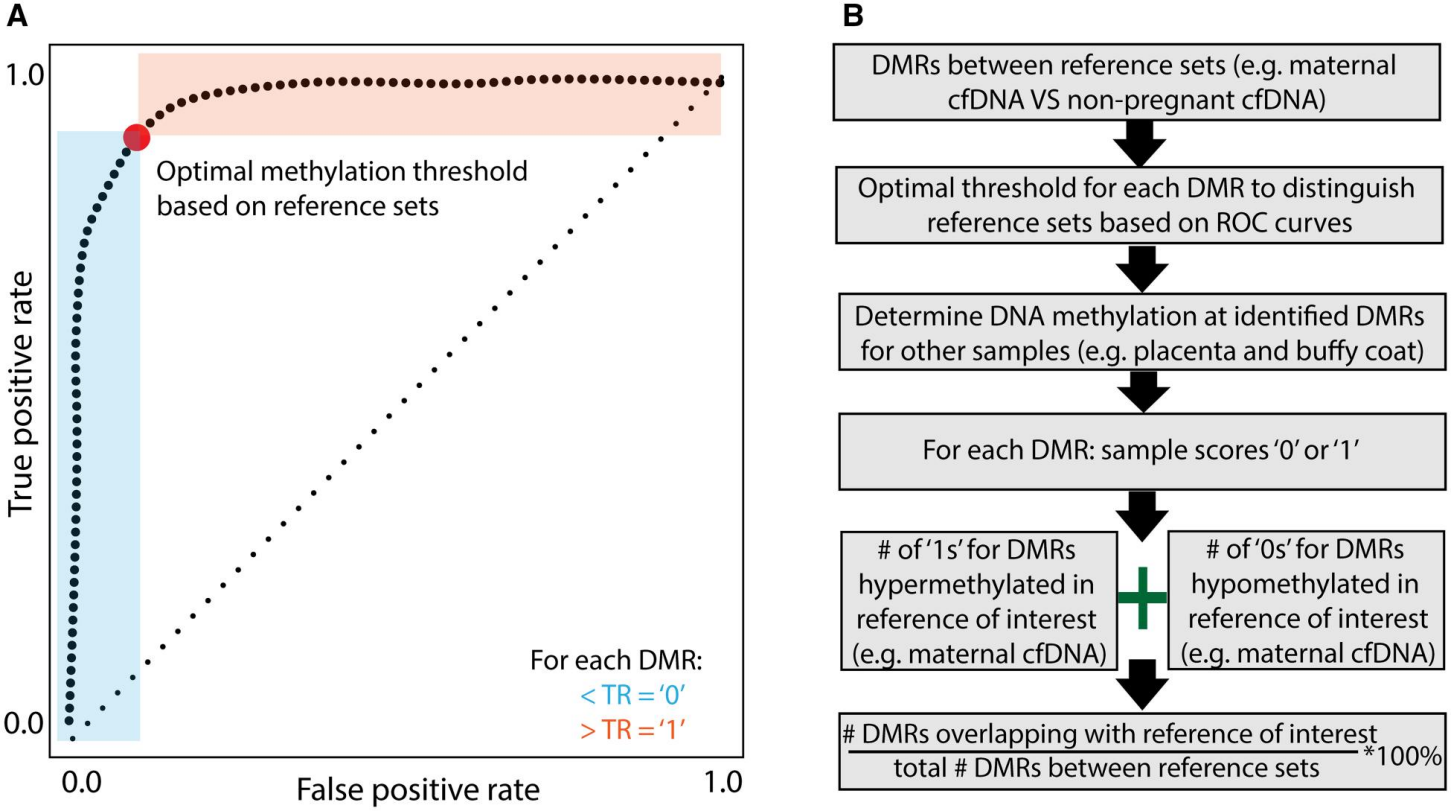
**Obtaining a cumulative methylation score based on a binary score for each differentially methylated region (DMR) of interest.** (**A**) Receiver operating characteristic curves were generated for each individual DMRs identified between reference sets of interest to determine the optimal methylation threshold that best distinguished both groups (e.g. maternal cell-free (cf)DNA compared to cfDNA from non-pregnant women). This threshold is defined based on the highest combination of the sensitivity (*Y*-axis) and specificity (*X*-axis is 1-specificity). (**B**) Workflow: first, DMRs between reference sets were identified (e.g. between maternal cfDNA and cfDNA from non-pregnant women). The calculated optimal threshold (A) was applied to all other samples of interest (e.g. placenta tissue and cell types, and buffy coat samples) leading to a binary methylation score for each DMR for these samples of interest. Samples with methylation levels below the threshold for the specific DMR scored ‘0’, and samples with methylation levels above the threshold for the specific DMR scored ‘1’. The cumulative methylation score for a specific sample (e.g. placenta or buffy coat) consists of the total number of DMRs above the set threshold (hypermethylated) that were also hypermethylated in the reference of interest (e.g. maternal cfDNA), together with the total number of DMRs for which the methylation level is below the set threshold (hypomethylated) that were also hypomethylated in the reference of interest. The total number of DMRs that overlap with the reference of interest, divided by the total number of DMRs between the reference sets is the proportion of overlapping DMRs.

To explore the tissue or cell type of origin of identified DMRs, we developed a binary methylation score for each DMR. Receiver operating characteristic curves of reference sets were used to calculate the optimal DNA methylation threshold (using the ‘scikit-learn’ package in Python; Pedregosa *et al.*, 2011) that best distinguished the two reference sets for each individual DMR (Fig. 1A). We compared this threshold to DNA methylation of that specific region in other tissues. Samples with a DNA methylation level above the threshold for the specific DMR scored ‘1’, and samples with a DNA methylation level below the threshold for the specific DMR scored ‘0’, leading to a binary score for each DMR (Fig. 1B). A cumulative methylation score was subsequently generated for each sample of interest based on the binary scores for all identified DMRs. For example, for identified DMRs that were hypermethylated in first-trimester cfDNA as compared to cfDNA of non-pregnant women, we counted, in each individual placenta and buffy coat sample, how many of these DMRs had a DNA methylation level of this specific region above the established threshold. Likewise, for all identified DMRs that were hypomethylated in first-trimester cfDNA as compared to cfDNA of non-pregnant women, we counted, in each individual placenta and buffy coat sample, how many of these DMRs had a DNA methylation level of this specific region below the established threshold. Together, they formed the proportion of DMRs in first-trimester maternal cfDNA that, based on the binary score, overlaps with DNA methylation in placenta and buffy coats, which is another major source of cfDNA (Moss *et al.*, 2018) (Fig. 1B).

Next, methylation of the *RASSF1* promoter, a specific placental DNA methylation marker (Zejskova *et al.*, 2010), and methylation of our previously identified top-ranked placental-specific DMRs were compared between collection periods of cfDNA using an unpaired *t*-test standardized for the number of RPM. These top-ranked DMRs were previously identified in first-trimester cfDNA and are related to *TMEM240*, *DHRS3*, and a CpG island upstream of *PCMTD2* (van Vliet *et al.*, 2025a).

Finally, we determined the presence of specific placental DNA methylation markers associated with the duration of gestation in cfDNA. We calculated the Pearson correlation score for each DMR identified between first trimester and term placental tissues and SCTs/CTBs with gestational age in all cfDNA samples. A Pearson correlation score ≥0.7 was used to define a positive association. A cumulative methylation score was calculated for cfDNA samples based on methylation levels of placental DMRs positively associated with gestational age in cfDNA. High DNA methylation levels for identified placental DMRs in hematopoietic cells can mask the detection of these placental DMRs in cfDNA, since maternal blood cells account for the majority of cfDNA fragments (Moss *et al.*, 2018). Therefore, we additionally selected DMRs between first trimester and term placental tissues that were hypomethylated in buffy coat samples (≥80% of samples scored ‘0’ based on the binary score) to perform targeted analyses in maternal cfDNA.

Population statistics are presented as absolute numbers and percentages (%) for categorical variables and as medians and interquartile ranges for continuous variables.

## Results

### Technical performance

An overview of the samples and their technical performance is provided in Supplementary Table S1. For cfDNA collected in the first and second trimester or at delivery, 9 out of 10 samples per collection period passed quality control. DNA methylation profiles were established for all third trimester cfDNA samples, all bulk placental tissues, and all specific placental cell types isolated using LCM.

### Methylation differences in maternal cfDNA during gestation

**Figure 2.**
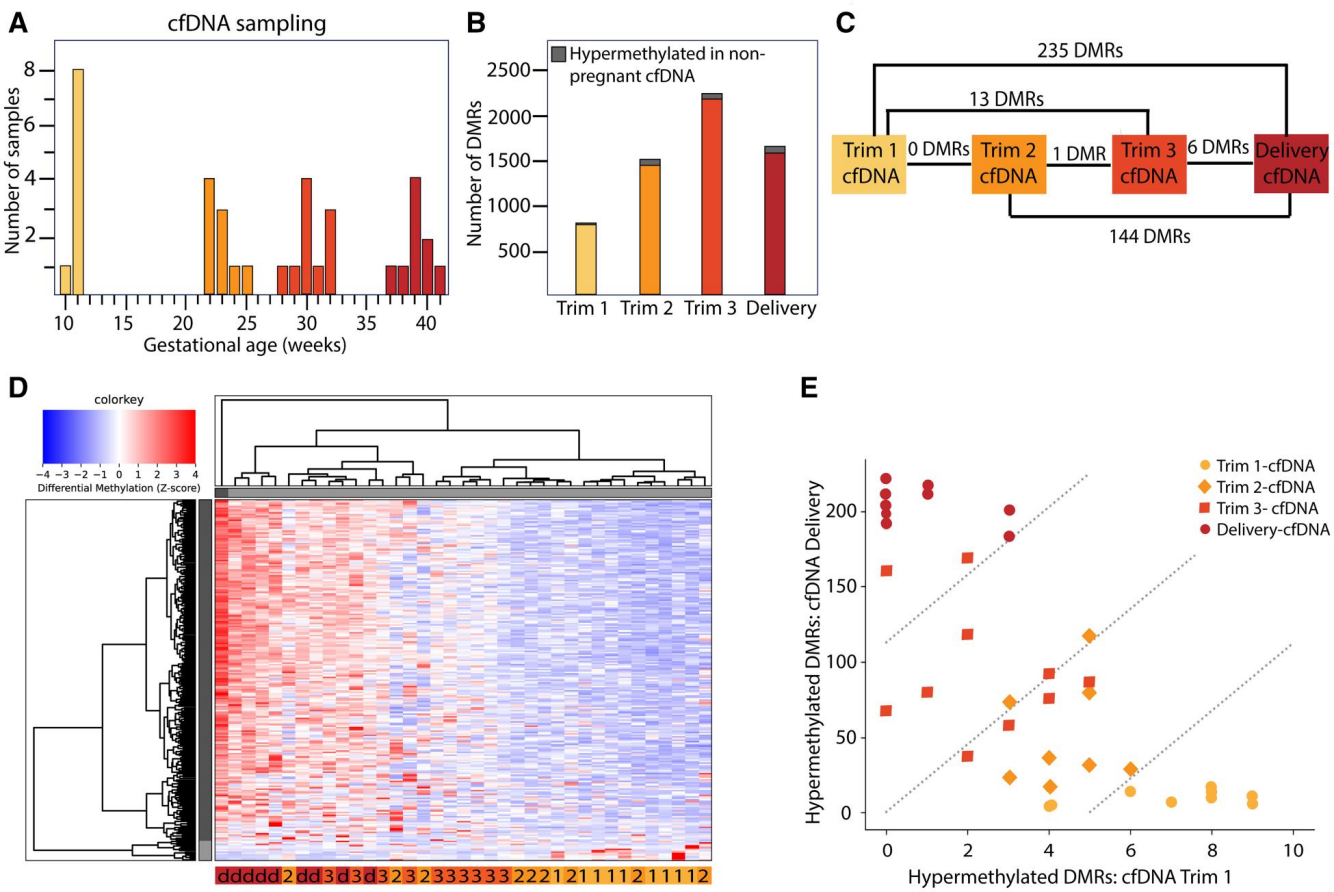
**DNA methylation in maternal cell-free (cf)DNA related to the duration of gestation.** (**A**) Gestational age at the time of cfDNA collection. (**B**) Number of identified differentially methylated regions (DMRs) between cfDNA collected at different periods during pregnancy and cfDNA from non-pregnant women. The majority of DMRs were hypermethylated in maternal cfDNA, with a small proportion of DMRs hypermethylated in cfDNA from non-pregnant women (gray). (**C**) Number of identified DMRs between cfDNA collected in different trimesters and at delivery. (**D**) Heatmap visualizing unsupervised hierarchical clustering of maternal cfDNA samples shows general clustering of samples collected during the same and consecutive time points. Selected autosomal DMRs were identified between cfDNA collected in the first trimester (Trim) and cfDNA collected at delivery (d) with a fold change ≥2. The three trimesters are depicted as 1, 2, and 3. (**E**) Cumulative methylation score based on DMRs between first trimester and delivery cfDNA for all maternal cfDNA samples. For each sample, the cumulative number of ‘1s’ for DMRs hypermethylated in first-trimester cfDNA are on the *x*-axis, and the cumulative number of ‘1s’ for DMRs hypermethylated in cfDNA collected at delivery are on the *y*-axis (Materials and methods).

The baseline characteristics of the pregnant women included in this study are depicted in Table 1. Plasma samples for cfDNA isolation were collected at 10 + 4–11 + 6 weeks (n = 9), 22 + 0–25 + 6 weeks (n = 9), and 28 + 6–32 + 6 weeks (n = 10) of gestation for the different trimesters. Sampling at delivery occurred between 37 + 4 and 41 + 0 weeks of gestation (n = 9) (Fig. 2A, Supplementary Table S1).

**Table 1.** Baseline characteristics of pregnant women with cell-free (cf) DNA samples (n = 10).

**Age at conception (years)**	32.0 (30.5–35.4)
**BMI at study entry**	25.3 (23.8–25.8)
**Country of birth**	
The Netherlands	8 (80%)
Europe, other	2 (20%)
**Nulliparous**	5 (50%)
**Periconceptional smoking**	1 (10%)
**Mode of conception**	
Spontaneous	5 (50%)
IVF/ICSI	5 (50%)
**Folic acid supplement use**	10 (100%)
Started before conception	9 (90%)
**Education level**	
Intermediate	6 (60%)
High	4 (40%)
**Male fetus**	4 (40%)
**Gestational age at birth**	39 + 2 (39 + 1–40 + 3)
**Birth weight (g)**	3655 (3458–3825)
**Birth weight (percentile)**	66 (54–79)

Categorical variables are described as absolute numbers and percentages (%). Continuous variables are described as medians and interquartile ranges. IVF/ICSI, *in vitro* fertilization/intracytoplasmic sperm injection.

In comparison to cfDNA from non-pregnant women, the number of identified DMRs in maternal cfDNA increased over the three trimesters, from 798 in the first trimester, 1494 in the second trimester, to 2163 DMRs in the third trimester, whereas 1600 DMRs were identified in cfDNA collected at delivery (Fig. 2B, Supplementary Table S2). The majority of the identified DMRs were hypermethylated in cfDNA collected during pregnancy as compared to cfDNA from non-pregnant women, with only 11 (1.4%), 80 (5.4%), 89 (4.1%), and 100 (6.3%) DMRs hypermethylated in cfDNA from non-pregnant women in the three trimesters or at delivery, respectively (Fig. 2B, Supplementary Table S2). The numbers of DMRs identified between the trimesters and delivery were considerably lower, ranging from no DMRs between the first and second trimester, to 235 DMRs between the first trimester and delivery (Fig. 2C, Supplementary Table S3). Additionally, only one DMR was found between the second and third trimester, 6 DMRs were found between the third trimester and delivery, 13 DMRs were identified between the first and third trimester, and 144 DMRs were found between the second trimester and delivery (Fig. 2C, Supplementary Table S3).

To study the course of identified DMRs during gestation, Fig. 2D shows an unsupervised hierarchical clustering analysis for DMRs identified in cfDNA between the first trimester and delivery. This shows a gradual change in DNA methylation for identified DMRs related to the duration of gestation, with an overall clustering of samples collected during the same time periods and in consecutive trimesters. Comparable results for these DMRs were obtained using a cumulative methylation score (Materials and methods, Fig. 1) when distinguishing overlap with DMRs specifically hypermethylated in cfDNA collected in the first trimester or at delivery (Fig. 2E, Supplementary Table S4), as well as for DMRs identified in cfDNA between non-pregnant women and cfDNA collected at delivery (Supplementary Fig. S1).

### DNA methylation differences in placental tissues during gestation

**Figure 3.**
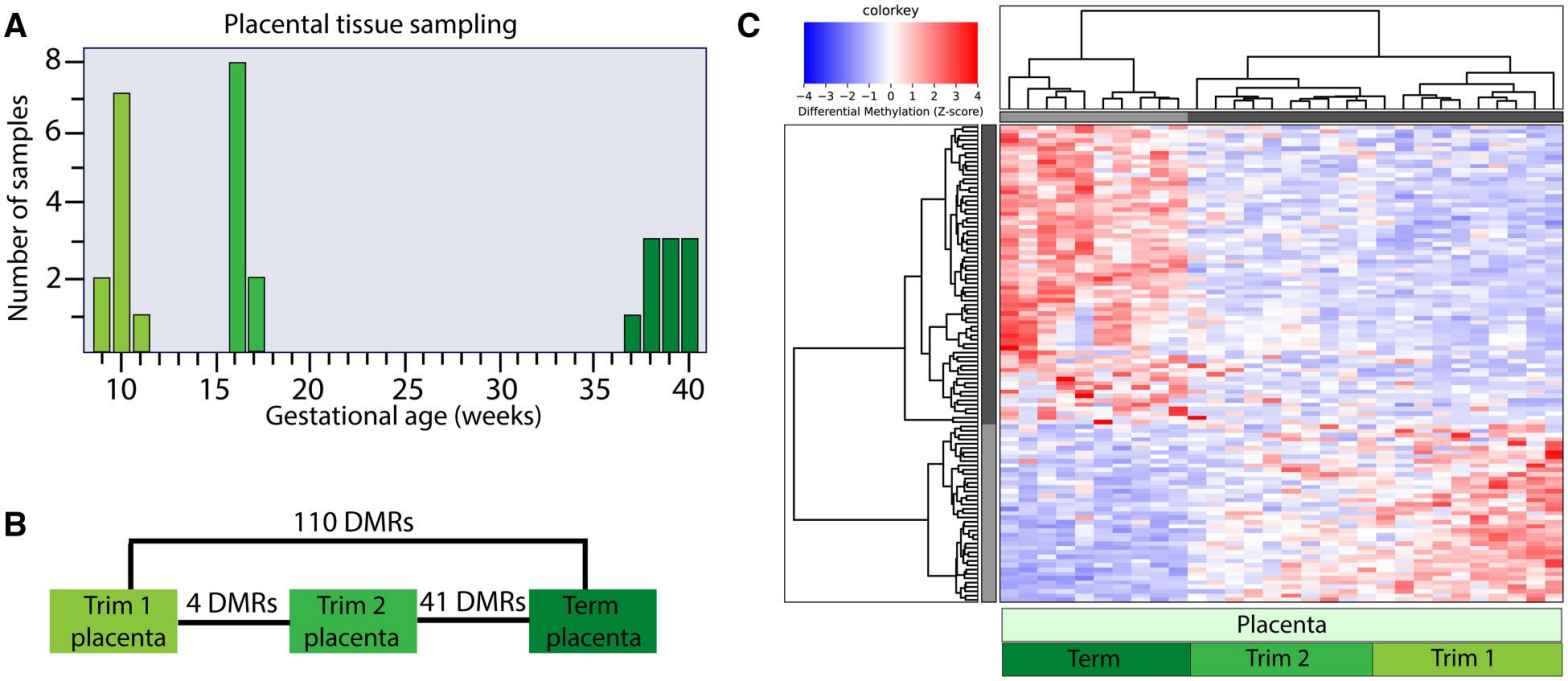
**DNA methylation in placental tissues in relation to the duration of gestation.** (**A**) Gestational age at the time of placental tissue collection. (**B**) Number of identified differentially methylated regions (DMRs) between placental tissues collected in the first and second trimester (Trim) and after term delivery. (**C**) Heatmap visualizing unsupervised hierarchical clustering of second-trimester placental tissues between first trimester and term placental tissues. Selected autosomal DMRs were identified between first trimester and term placental tissues with a fold change ≥2.

Next, we investigated differences in DNA methylation between placental tissues collected at different periods during gestation. Placental tissues were collected between 9 + 2–12 + 2 and 16 + 0–17 + 6 weeks of gestation in the first and second trimester, respectively, and after-term deliveries between 37 + 4–40 + 6 weeks of gestation (all n = 10) (Fig. 3A, Supplementary Table S1). We identified 110 autosomal DMRs between first trimester and term placentas, 4 DMRs between first- and second-trimester placentas, and 41 DMRs between second trimester and term placentas (Fig. 3B, Supplementary Table S5). Unsupervised hierarchical clustering analysis for DMRs identified between first trimester and term placental tissues showed that placental tissues from second-trimester cluster between first trimester and term placental tissues (Fig. 3C), indicating a gradual, progressive change in placental DNA methylation during gestation.

### DNA methylation differences in relation to duration of gestation and between placental cell types

**Figure 4.**
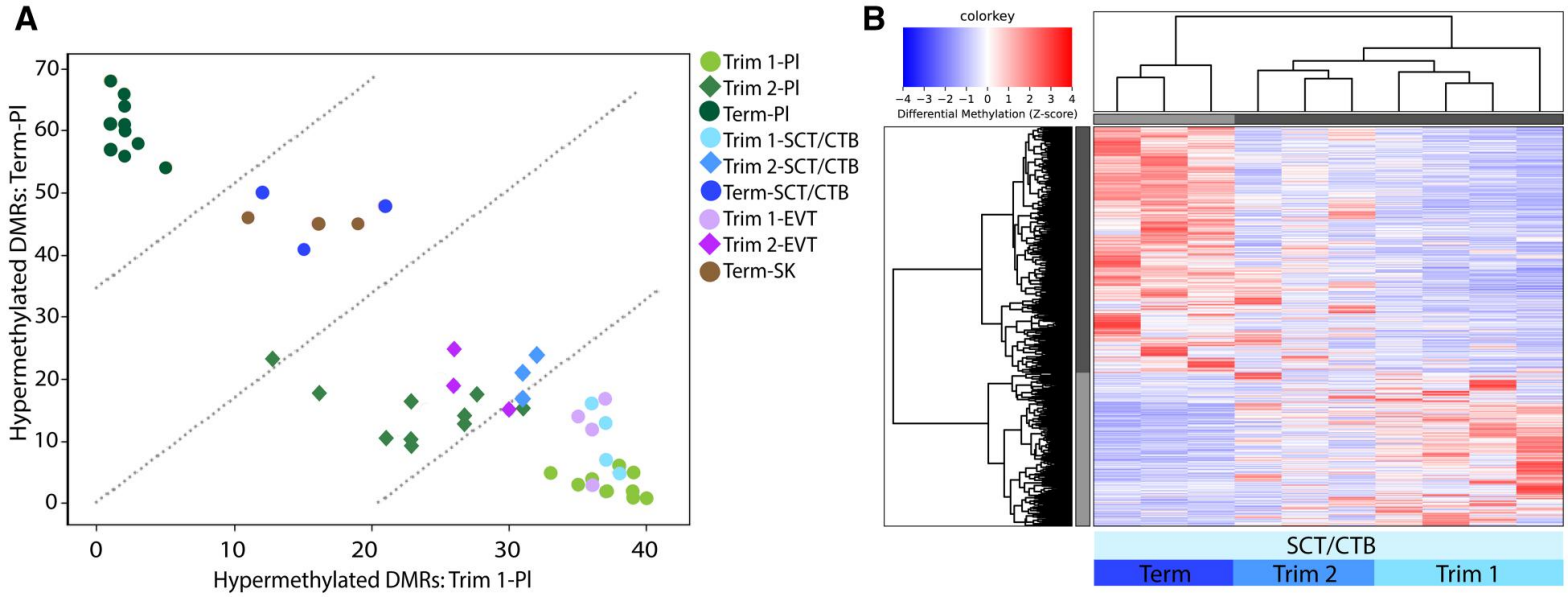
**DNA methylation associated with the duration of gestation in trophoblasts.** (**A**) Cumulative methylation scores for placental cell types isolated after laser capture microdissection (LCM), based on methylation of differentially methylated regions (DMRs) identified between bulk term placenta (Term-Pl) and first-trimester placenta (Trim 1-Pl). For each LCM sample, the cumulative number of ‘1s’ for DMRs hypermethylated in first-trimester placental tissues are on the *x*-axis, and the cumulative number of ‘1s’ for DMRs hypermethylated in term placental tissues are on the *y*-axis. The different trophoblast populations show the greatest overlap with placental tissues collected at a comparable gestational age. (**B**) Heatmap visualizing unsupervised hierarchical clustering of second-trimester SCTs/CTBs between first trimester and term SCTs/CTBs. Selected autosomal DMRs were identified between first trimester and term SCTs/CTBs with a fold change ≥2. Pl, (bulk) placenta; SCT/CTB, syncytiotrophoblasts and cytotrophoblasts; SK, syncytial knotting; EVT, extravillous trophoblasts.

We further zoomed in on different placental cell types to explore the cell type of origin of DNA methylation and monitor potential changes of contribution of these cell types in bulk placental tissues and maternal cfDNA. Using LCM, we isolated SCTs and CTBs from first (n = 4) and second (n = 3) trimester, and from term (n = 3) placentas, EVTs from first (n = 4) and second (n = 3) trimester placentas, and SK (n = 3) from term placentas. Additionally, maternal-originated endometrial epithelium and spiral arteries from first (both n = 2) and second (both n = 1) trimester placentas were isolated. Using a cumulative methylation score based on binary scores for all DMRs between first trimester and term bulk placental tissues, we found that the methylation profiles of different trophoblast populations (SCTs/CTBs, EVTs, and SK) are most comparable to the methylation profiles in bulk placental tissues collected at a comparable gestational age (Fig. 4A). For first- and second-trimester endometrial epithelium and spiral arteries, a relationship between identified placental DMRs and gestational age was not observed (Supplementary Fig. S2 and Table S6), so these cell types cannot explain the gestational age-associated differences observed in bulk placental DNA. Additionally, we identified 3122 DMRs between first trimester and term SCTs/CTBs, 1320 DMRs between first- and second-trimester SCTs/CTBs, and 1911 DMRs between second trimester and term SCTs/CTBs (Supplementary Table S7). Using DMRs between first trimester and term SCTs/CTBs, second-trimester SCTs/CTBs cluster in between in an unsupervised hierarchical clustering analysis (Fig. 4B). Both showed that DNA methylation changes related to gestational age are also observed in trophoblasts, indicating a gradual, progressive change in DNA methylation of trophoblast cells during placental development.

Despite the large overlap between different trophoblast populations from the same trimester for DMRs identified in bulk placental tissues related to the duration of gestation (Fig. 4A), we identified numerous differences in DNA methylation between different trophoblast populations within the same trimester. We identified 601 and 1610 DMRs between SCTs/CTBs and EVTs in the first and second trimester, respectively, and 558 DMRs between term SCTs/CTBs and SK (Supplementary Fig. S3 and Table S8). These cell type-specific DNA methylation profiles may be used to evaluate the contribution of these specific cell types to the methylation signature in maternal cfDNA.

### Origins of DMRs in maternal cfDNA

**Figure 5.**
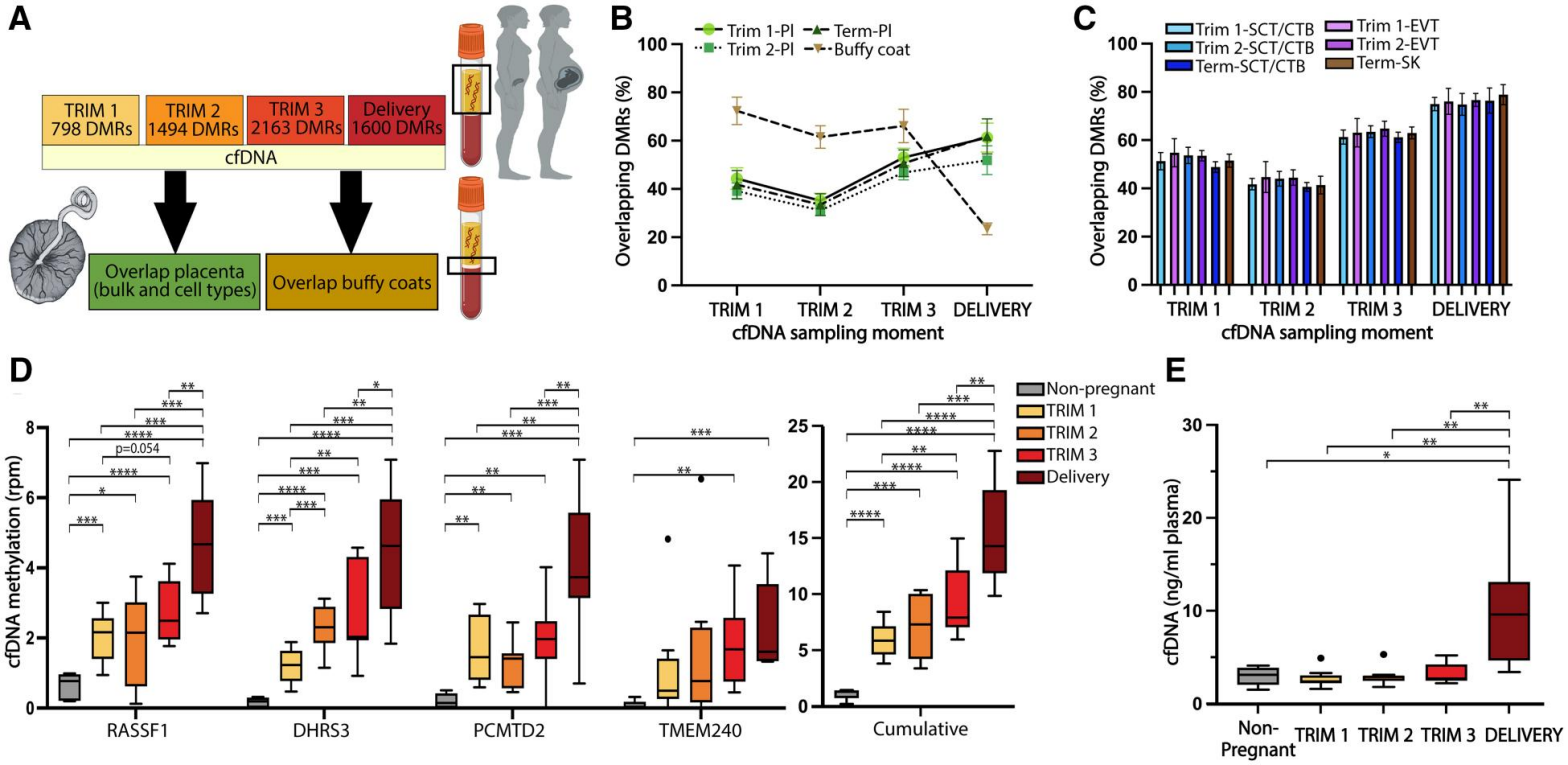
**(Placental) origin of differentially methylated regions (DMRs) identified in maternal cell-free (cf)DNA as compared to cfDNA from non-pregnant women.** (**A**) Overlap of DMRs identified in maternal cfDNA across gestation as compared to cfDNA from non-pregnant women was studied in placental bulk tissues, specific placental cell types, and buffy coats. (**B, C**) The proportion of DMRs identified in maternal cfDNA in the different trimesters (Trims) and at delivery as compared to cfDNA from non-pregnant women, that overlap with DNA methylation in placental tissues and buffy coats (B), and with different trophoblast populations (C) based on our cumulative methylation scores. The means and SDs are depicted. (**D**) Box plot showing methylation of the RASSF1 promoter, methylation of three previously identified placental-specific methylation markers (DMRs related to DHRS3, PCMTD2, and TMEM240), and methylation of these four markers combined in cfDNA across gestation. *Y*-axis depicts the normalized number of reads per million reads, see also Supplementary Table S10. (**E**) Box plot showing the cfDNA concentration per milliliter of plasma in samples from non-pregnant women and from pregnant women for each trimester and at delivery. No differences were found in the cfDNA concentration between cfDNA from non-pregnant women and cfDNA collected in the three trimesters during pregnancy. *P ≤ 0.05, **P ≤ 0.01, ***P ≤ 0.001, ****P ≤ 0.0001, all based on *t*-test. Pl, (bulk) placenta; SCT/CTB, syncytiotrophoblast/cytotrophoblast; EVT, extravillous trophoblast; SK, syncytial knotting.

Besides the presence of placental-originated cfDNA in maternal cfDNA, physiological pregnancy-induced changes in other tissues contributing to maternal cfDNA could be important drivers of observed DMRs in maternal cfDNA. To explore the (placental) origin of DMRs identified in maternal cfDNA, we therefore studied the overlap with DNA methylation in bulk placental tissues, placental cell types, and maternal blood buffy coats, another major source of cfDNA (Moss *et al.*, 2018), for DMRs identified between pregnant and non-pregnant women (Fig. 5A). Using our cumulative methylation scores based on a binary score for each identified DMR (Materials and methods, Fig. 1A and B), the mean proportion of identified DMRs in maternal cfDNA compared to cfDNA from non-pregnant women that overlapped with DNA methylation in our placental tissue samples increased from 41.8% (SD 4.97) in the first trimester, to 59.9% (SD 7.73) at delivery. Comparable results were found for placental tissues collected at different periods during gestation (Fig. 5B). Conversely, the mean proportion of DMRs that overlapped with DNA methylation of buffy coats was 72.4% (SD 5.68) in first-trimester cfDNA, and this decreased with advancing gestational age, especially after the third trimester, to 25.4% (SD 2.24) in cfDNA at delivery (Fig. 5B, Supplementary Table S9A–D).

Zooming in on different placental cell types, a comparable course in overlapping DMRs as for the bulk placental samples was found for the different types of trophoblasts collected in each trimester (Fig. 5C). The mean proportion of identified DMRs in maternal cfDNA that overlapped with DNA methylation of trophoblasts was higher as for bulk placenta and increased from 52.4% (SD 3.8) in the first trimester to 76.2% (SD 3.9) at delivery (Supplementary Table S9A–D, Fig. 5C). Conversely, there was considerably less overlap with DNA methylation of spiral arteries and the endometrial epithelium throughout gestation (Supplementary Fig. S4 and Table S9A–D). Based on DMRs identified between different trophoblast populations, we did not specifically distinguish DNA methylation markers from a specific trophoblast population over another trophoblast population (EVTs, SCT/CTBs, or SK) in maternal cfDNA (Supplementary Fig. S5).

As a measure of the placental-originated fraction in total cfDNA, we investigated the methylation of the *RASSF1* promoter, a placental-specific marker, during gestation (Zejskova *et al.*, 2010) (Supplementary Table S10). As compared to cfDNA from non-pregnant women, *RASSF1* promoter methylation was higher in cfDNA collected in the different trimesters and at birth. In first- and second-trimester cfDNA, the mean *RASSF1* promoter methylation increased with a fold change (FC) of 3.07 (*P* = 0.0008) and 3.01 (*P* = 0.034), respectively (Fig. 5D). No difference in *RASSF1* methylation was found between first- and second-trimester cfDNA (mean FC = 0.98, *P* = 0.93). As compared to cfDNA from non-pregnant women, the mean *RASSF1* promoter methylation increased with a FC of 4.20 (*P* < 0.0001) in third trimester cfDNA, and a FC of 7.06 (*P* < 0.0001) in cfDNA collected at delivery. Between first and third trimester cfDNA, *RASSF1* promoter methylation increased with an FC of 1.37 (*P* = 0.054). As compared to cfDNA collected in the three different trimesters, the mean *RASSF1* promoter methylation was higher in cfDNA at delivery (FC = 2.30, *P* = 0.0002; FC = 2.35, *P* = 0.0001; FC = 1.68, *P* = 0.0031, respectively) (Fig. 5D). For our previously identified, additional, top-ranked placental-specific DMRs related to *TMEM240, DHRS3*, and a CpG island upstream of *PCMTD2* (van Vliet *et al.*, 2025a), a comparable gestational age-related increase in DNA methylation was found (Fig. 5D, Supplementary Fig. S6 and Table S10). The cumulative methylation of these four markers shows a clear, consequent increase with advancing gestational age (Fig. 5D).

The increase in placental-derived cfDNA was most apparent at delivery, particularly considering the increased total cfDNA concentration in plasma at delivery. While the total cfDNA concentration did not differ between non-pregnant women and the three trimesters, the total cfDNA concentration per milliliter of plasma was about 3–4 times higher at delivery as compared to the other sampling periods (*P*-values between 0.001 and 0.019) (Fig. 5E).

### Presence of trimester-specific placental DMRs in maternal cfDNA

**Figure 6.**
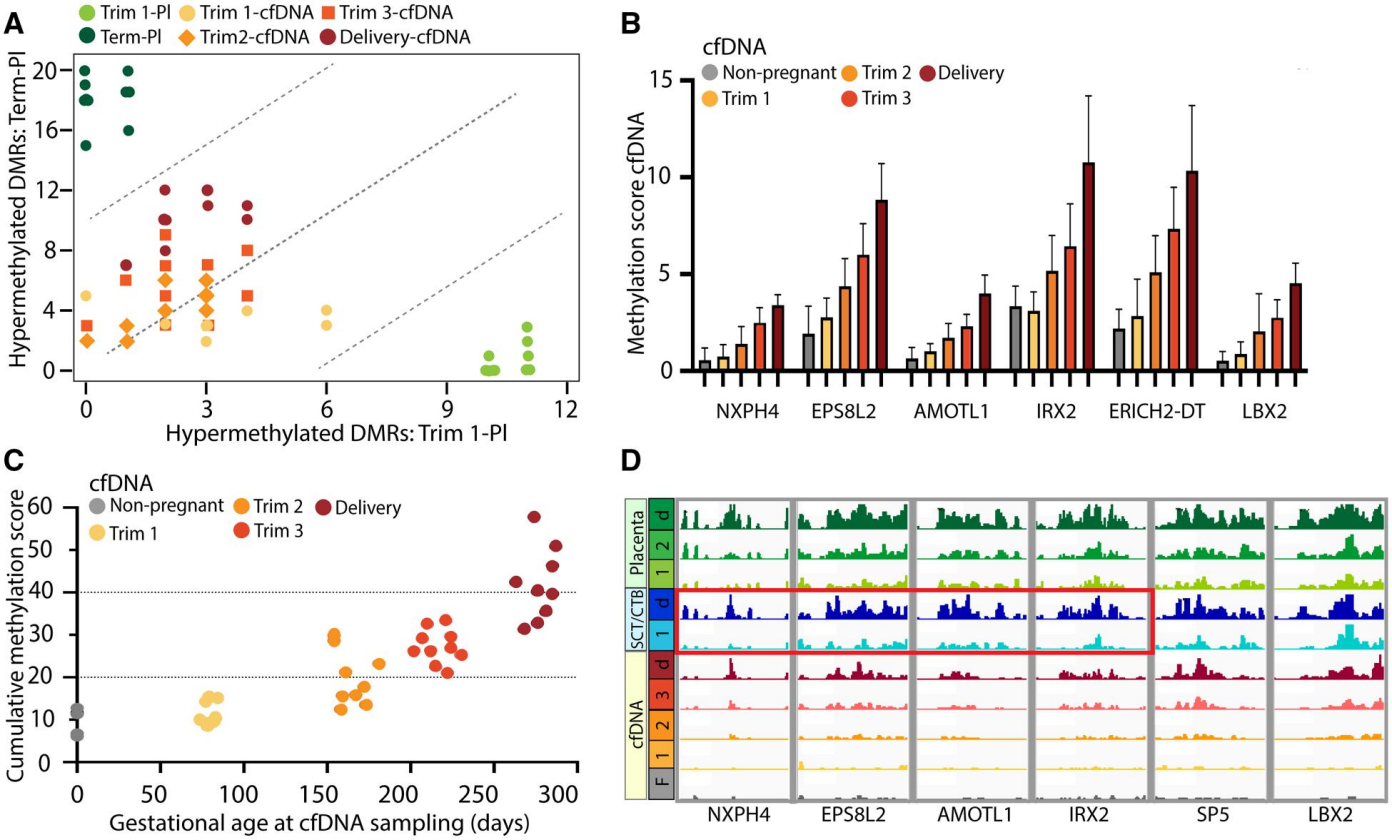
**Differentially methylated regions (DMRs) associated with gestational age in placental tissues and in maternal cell-free (cf)DNA.** (**A**) The overlap of DMRs identified between first trimester and term placental tissues was studied in maternal cfDNA collected in the different trimesters and at delivery using a cumulative methylation score. Only DMRs with low DNA methylation levels in buffy coat samples were selected (n = 32). The *Y*-axis represents overlap with DMRs hypermethylated in term placental tissues, and the *X*-axis represents overlap with DMRs hypermethylated in first-trimester placental tissues. Each dot represents one sample. (**B**) Mean methylation score in cfDNA for six DMRs between first trimester and term placental tissues, that are positively associated with gestational age in cfDNA (Pearson correlation score ≥0.7). The error bars depict the SDs. (**C**) Cumulative methylation score for each cfDNA sample, based on the six identified placental DMRs associated with gestational age in cfDNA. (**D**) Average gene tracks for the six identified placental DMRs associated with gestational age in cfDNA. The first four DMRs were also found between the first trimester and term SCTs/CTBs (outlined in red). SCT/CTB, syncytiotrophoblasts/cytotrophoblasts; F, non-pregnant women; 1, first trimester; 2, second trimester; 3, third trimester; d, delivery.

Besides the increase in methylation of universal placental-specific markers during gestation, we further investigated the presence in maternal cfDNA of DNA methylation profiles that were not found in all placental tissues, but that were related to gestational age in placental tissue. Most gestational age-specific DMRs identified in placental tissues were not specifically present in cfDNA collected at a comparable gestational age. Based on a cumulative methylation score for all DMRs identified between first trimester and term placental tissues, maternal cfDNA collected at different periods in pregnancy showed largely comparable overlap (Supplementary Fig. S7 and Table S11). However, a change in placental DNA methylation could be masked in maternal cfDNA if methylation of these regions is high in hematopoietic cells, which account for the majority of cfDNA fragments. We therefore selected DMRs identified between first trimester and term placental tissues with low DNA methylation levels in buffy coats. Of 110 DMRs, 32 DMRs were selected which were hypomethylated in ≥80% of buffy coat samples based on the binary scores. Of those, 11 were hypermethylated in first-trimester placental tissues, and 21 were hypermethylated in term placental tissues (Supplementary Table S12). We observed a clear increase in DNA methylation in cfDNA across gestation of DMRs specifically hypermethylated in term placental tissues, while a decrease in DNA methylation of DMRs hypermethylated in first trimester was less clear (Fig. 6A).

When all individual DMRs were investigated, six DMRs identified between first trimester and term placental tissues showed high correlations with gestational age in cfDNA (Pearson correlation ≥0.7) (Supplementary Table S13). These DMRs were related to *EPS8L2*, *AMOTL1*, *NXPH4*, *SP5*, *LBX2*, and *IRX2* (Fig. 6B) and all had low DNA methylation levels in buffy coat samples. Moreover, Fig. 6C shows a cumulative methylation score based on these 6 DMRs for all cfDNA samples. After investigating individual DMRs identified between first trimester and term SCT/CTBs, five DMRs were associated with gestational age in cfDNA (Pearson correlation ≥0.7) (Supplementary Table S14). Four of these DMRs overlapped with the six DMRs identified in bulk placental tissues, and were related to *EPS8L2*, *AMOTL1*, *NXPH4*, and *IRX2* (Fig. 6D).

This indicates that, besides the detection of universal placental-specific markers, also placental methylation marks related to the duration of gestation, particularly those originating from SCT/CTBs, could be identified in maternal cfDNA. A prerequisite for the detection of these placental DNA methylation markers in maternal cfDNA is hypomethylation for these DMRs in hematopoietic cells.

## Discussion

We used MeD-seq to longitudinally investigate genome-wide maternal cfDNA methylation across pregnancy, including each trimester and at birth. To understand and identify which cfDNA methylation markers originate from placental tissue, we investigated DNA methylation in placental tissues and specific placental cell types in each trimester.

We showed a gradual change in DNA methylation across gestation in maternal cfDNA and in placental tissues and trophoblasts. With advancing gestational age, we observed an increase in DMRs identified in maternal cfDNA that overlapped with DNA methylation of placental tissues, particularly in trophoblast populations, and an increase in DNA methylation of known placental-specific markers. These findings suggest that the identified DMRs in maternal cfDNA during gestation are largely attributable to the known increasing placental-derived cfDNA fraction especially after 20 weeks of gestation (Sun *et al.*, 2015; Del Vecchio *et al.*, 2021; Zaki-Dizaji *et al.*, 2023), and underline the trophoblast-origin of the placental signature in maternal cfDNA. We did not specifically distinguish DNA methylation markers from a specific trophoblast population over another trophoblast population (Supplementary Fig. S5), which could indicate that the different trophoblast populations all contribute to the methylation signature in maternal cfDNA.

Additionally, we found a large overlap of DMRs identified in maternal cfDNA in all three trimesters with DNA methylation in buffy coats, suggesting an increased immune response during pregnancy (van Vliet *et al.*, 2025a). This also underlines that there are different tissue-of-origins driving the DMRs that are identified in maternal cfDNA, and this should be taken into account when interpreting cfDNA methylation differences related to pregnancy. Since an increase in placental-originated cfDNA fragments will lead to a relatively decreased contribution of cfDNA fragments from maternal blood cells to total maternal cfDNA, this can explain the decrease in overlap with DMRs found in buffy coats during gestation.

The clear increase during gestation in methylation of our previously identified placental-specific DMRs underlines their placental-origin. A combined methylation score for these different placental-specific markers in addition to *RASSF1* promoter methylation could potentially be used in the determination of the placental-fraction in total maternal cfDNA, for which multiple methods exist (Nygren *et al.*, 2010).

Finally, we focused on 32 DMRs identified between first trimester and term placental tissues that were hypomethylated in buffy coat samples. Methylation markers specifically present in term placental tissues showed increasing methylation levels in cfDNA across gestation, while a decrease in first-trimester placental methylation markers was less clear. Six DMRs hypermethylated in term as compared to first-trimester placental tissues showed the strongest correlation with gestational age in maternal cfDNA, four of which were also found in SCTs/CTBs. These DMRs were related to *NXPH4*, *EPS8L2*, *AMOTL1*, and *IRX2*. The preselection of markers identified directly in placental tissues supports the feasibility of identifying specific gestational age-related placental DNA methylation markers in maternal cfDNA. However, the difference in detection between first trimester and term placental methylation markers is likely due to the simultaneously increasing placental-derived cfDNA fraction, enhancing the detectability of placental DNA methylation markers with advancing gestational age.

The large differences in the number of identified DMRs between cfDNA from pregnant versus non-pregnant women, compared with the number of DMRs identified between trimesters suggest that most DNA methylation changes in cfDNA during pregnancy are minor compared to the DNA methylation changes invoked by the pregnancy itself, which are already present at the end of the first trimester. Moreover, to prevent the identification of false positive results, the MeD-seq pipeline uses the Bonferroni correction and a minimal FC of 2 for identified DMRs. This rather stringent approach combined with a relatively small difference in the placental-originated fraction between subsequent sampling periods could explain the lack of acquiring this threshold for most DMRs.

A ‘diluted’ placental signature in total cfDNA and/or specific methylation profiles of cfDNA fragments originating from maternal tissues can also impact the detectability of placental methylation in total cfDNA. For example, for regions highly methylated in maternal blood cells and thus in cfDNA, the change in average methylation of total cfDNA caused by changes in placental DNA methylation will often be below two and remain undetected. Focusing on regions with low methylation levels in leukocytes as compared to placental tissues, as done for the gestational age-specific placental markers and as done by De Borre *et al.* (2023), or an enrichment of placental-originated cfDNA based on the shorter fragment size of placental-originated cfDNA as compared to maternal-originated cfDNA (Lo *et al.*, 2021), could partly overcome this technical issue in future studies.

DMRs identified in placental tissues and cell types in relation to the duration of gestation are, as expected, related to genes with important roles in various cellular functions and developmental processes, such as the regulation of gene transcription and differentiation. Differentially methylated CpGs related to *NXPH4* and *LBX2* have previously been described in a study that developed a placental epigenetic clock (Lee *et al.*, 2019), whereas aberrant methylation and/or expression of *NXPH4*, *EPS8L2*, *AMOTL1*, *IRX2*, and *LBX2* have also been described in multiple types of cancers (Colas *et al.*, 2011; Huang *et al.*, 2020; Wang and Yu, 2024; Xu *et al.*, 2024; Zhang *et al.*, 2024). Similarities between the placenta and cancers in terms of DNA methylation have frequently been described and are suggested to play a role in mutual properties, including their invasive character, cell proliferation, and immune modulation (Costanzo *et al.*, 2018).

Apart from gestational age differences in DNA methylation driven by placental development and differentiation over time as also shown by others (Yuan *et al.*, 2021; Zhang *et al.*, 2021), the varying composition of different cell types likely plays a significant role in driving the observed differences. For example, the abundance of SCTs is higher than CTBs in term placenta as compared to first-trimester placenta (Mayhew and Barker, 2001). However, DNA methylation changes caused by differences in cell composition would likely also lead to methylation differences in cfDNA and could therefore be of great value in identifying placental-originated methylation differences in cfDNA. This is supported by our data suggesting that different trophoblast types (SCTs/CTBs, EVTs, SK) have unique DNA methylation patterns and all contribute to the methylation signature in maternal cfDNA.

Strengths of the current study include that we longitudinally studied genome-wide maternal cfDNA methylation dynamics across gestation, which has been largely understudied thus far. DNA methylation profiles in placental tissues and cell types across gestation were correlated with DNA methylation in maternal cfDNA. Moreover, the high genome-wide coverage of MeD-seq leads to the identification of novel methylation markers, and our stringent approach minimizes false positive results. On the other hand, our rather strict approach could also lead to fewer identified regions and more false negative results. Furthermore, owing to the substantial efforts required for the isolation of placental cell types, our sample size for specific placental cell types was relatively small. We also did not account for potential confounders such as smoking, maternal age, and ethnicity, since we had no access to detailed information about the participants’ characteristics for first- and second-trimester placental tissues or non-pregnant women. However, the longitudinal sampling of maternal cfDNA minimizes bias in our cfDNA analyses and we used data from specific placental cell types to validate our results from bulk placental tissues. Finally, the fragmented nature of cfDNA could impact the coverage of the genome.

Although the genome-wide cfDNA methylome during pregnancy is largely understudied, cfDNA holds great promise for studying epigenetic (placental) programming during pregnancy and to develop clinically relevant biomarkers to further improve obstetrical care. We show the possibility to detect robust placental methylation markers in maternal cfDNA in relation to gestational age. Moreover, promising results have been reported recently indicating the feasibility of predicting the risk for preeclampsia by genome-wide cfDNA methylome profiling at an early pregnancy stage (De Borre *et al.*, 2023; He *et al.*, 2023). Also, the placental-derived fraction has been described to differ in pregnancies with preeclampsia (Carbone *et al.*, 2020; Khalil *et al.*, 2024). Both differences in the placental cfDNA fraction and actual DNA methylation differences in placental cells (or perhaps also in maternal tissues) (Stanley *et al.*, 2024), could jointly lead to improved biomarker development in future studies. Since cfDNA is already widely used for NIPT, in the future, cfDNA methylation profiling could possibly be combined with the current NIPT (Aerden *et al.*, 2024), moving forward to what we would call an epigenome-wide NIPT (Epi-NIPT) (van Vliet *et al.*, 2025a).

## Conclusion

This study increases our understanding of the maternal cfDNA methylome across gestation. We showed that most DNA methylation differences in maternal cfDNA across gestation are attributable to an increased placental fraction. Additionally, we identified specific gestational age-dependent placental DNA methylation marks in maternal cfDNA, paving the way for further research aimed at detecting placental methylation markers in maternal cfDNA. The study of cfDNA methylome profiling in relation to obstetrical complications warrants further study, for which this study could serve as a reference.

## Supplementary Material

gaaf011_Supplementary_Data

## Data Availability

All process data on which our results are based are available in the Supplementary Material. The MeD-seq datasets generated and/or analyzed during the current study are available in the National Center for Biotechnology Information (SRA database) with accession numbers PRJNA1108949 and PRJNA1147893 (see Supplementary Table S1).
