# Supplementary material for: Profiling (placental) DNA methylation in cell-free DNA across gestation: the Rotterdam Periconception Cohort
Source: Mol Hum Reprod. 2025 May 8;31(2):gaaf011. doi: 10.1093/molehr/gaaf011 (PMC12076144; doi:10.1093/molehr/gaaf011)
Supplement: gaaf011_Supplementary_Data [file gaaf011_supplementary_data.zip › Supplementary_data_figures_MHR.pdf]

Profiling (placental) DNA methylation in cell-free DNA across gestation: The Rotterdam Periconception Cohort

Authors: Marjolein M. van Vliet, Ruben G. Boers, Joachim B. Boers, Olivier J.M. Schäffers, Lotte E. van der Meeren, Joost Gribnau, Sam Schoenmakers, Régine P.M. Steegers-Theunissen

Supplementary information

Contents Page

Supplementary Figure S1: DNA methylation in cfDNA related to the duration of gestation

Supplementary Figure S2: DNA methylation associated with the duration of gestation in endometrial cells and spiral arteries

Supplementary Figure S3: DMRs identified between trophoblast populations collected at a comparable gestational age

Supplementary Figure S4: Overlap of DNA methylation of endometrial cells and spiral arteries with DMRs identified in maternal cfDNA

Supplementary Figure S5: DNA methylation in maternal cfDNA associated with specific trophoblast populations.

Supplementary Figure S6: Gene-tracks for three top-ranked placental-specific DMRs that were previously identified in cfDNA

Supplementary Figure S7: DNA methylation in maternal cfDNA for all (n=110) DMRs identified between first trimester and term placental tissues

Supplementary Tables are provided in separate Excel file

Supplementary Table S1: Overview of the used samples and their technical performance

Supplementary Table S2: Identified DMRs between cfDNA from non-pregnant women and cfDNA collected in the different trimesters and at birth

Supplementary Table S3: Identified DMRs between different sampling moments of cfDNA during pregnancy

Supplementary Table S4: Cumulative methylation score for all cfDNA samples, based on all DMRs identified between cfDNA from non-pregnant women and cfDNA collected at delivery

Supplementary Table S5: Identified DMRs between first and second trimester and term placental tissues

Supplementary Table S6: Cumulative methylation score for LCM samples, based on all DMRs identified between first trimester placental tissues and term placental tissues

Supplementary Table S7: Identified DMRs between first and second trimester and term SCTs/CTBs

Supplementary Table S8: Identified DMRs between different trophoblast populations collected in the first trimester (left), second trimester (middle), and in term placenta (right)

Supplementary Table S9: Cumulative methylation scores for placental tissues, cell types, and buffy coats, based on all DMRs identified between cfDNA from non-pregnant women and A) first trimester cfDNA, B) second trimester cfDNA, C) third trimester cfDNA, and D) cfDNA collected at delivery

Supplementary Table S10: *RASSF1* promoter methylation and methylation of three previous identified placental-specific DMRs for all cfDNA samples

Supplementary Table S11: Cumulative methylation score for maternal cfDNA samples, based on all DMRs identified between first trimester placental tissues and term placental tissues

Supplementary Table S12. Cumulative methylation score for maternal cfDNA samples, based on DMRs identified between first trimester placental tissues and term placental tissues that are hypomethylated in buffy coat samples

Supplementary Table S13: **Raw normalized reads for all cfDNA samples for all DMRs identified between first trimester placental tissues and term placental tissues.** Pearson correlation score is calculated to investigate the association with gestational age for each DMR.

Supplementary Table S14: **Raw normalized reads for all cfDNA samples for all DMRs identified between first trimester SCTs/CTBs and term SCTs/CTBs.** Pearson correlation score is calculated to investigate the association with gestational age for each DMR.

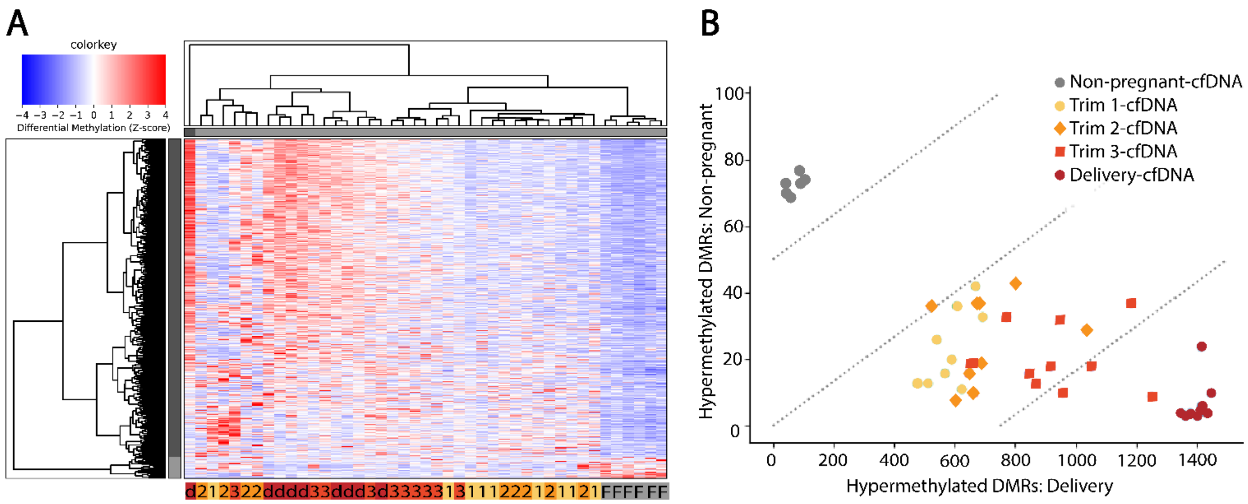

Supplementary Figure S1: DNA methylation in cfDNA related to the duration of gestation. A) Heatmap visualizing unsupervised hierarchical clustering of all cfDNA samples shows generally clustering of samples collected during the same and consecutive time points. Selected autosomal DMRs were identified between cfDNA collected in non-pregnant women (F) and cfDNA collected from pregnant women at delivery (d) with a fold change ≥2. The three trimesters are depicted as 1, 2, and 3. **B)** Cumulative methylation score based on DMRs between cfDNA from non-pregnant women and cfDNA collected at delivery for all cfDNA samples. For each sample, the cumulative number of '1s' for DMRs hypermethylated in cfDNA at delivery are on the x-axis, and the cumulative number of '1s' for DMRs hypermethylated in cfDNA from non-pregnant women are on the y-axis (Methods).

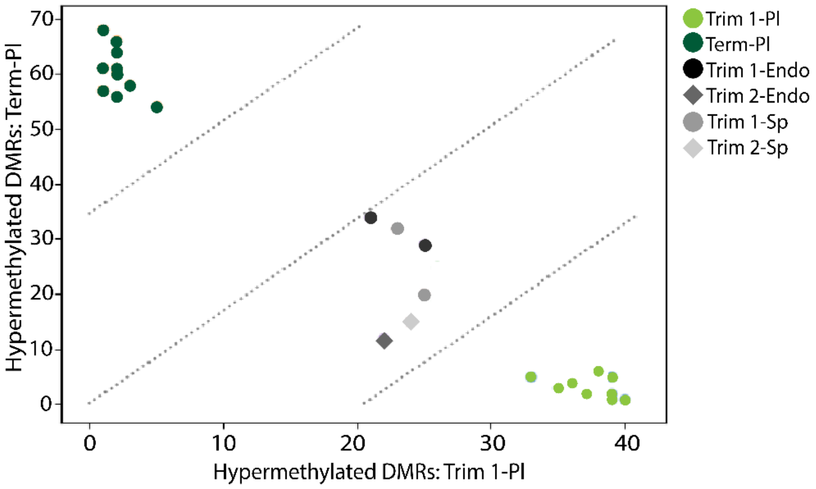

Supplementary Figure S2: DNA methylation associated with the duration of gestation in endometrial cells and spiral arteries. Cumulative methylation scores for endometrial cells and spiral arteries isolated after laser capture microdissection, based on methylation of DMRs identified between bulk term placenta (Term-Pl) and first trimester placenta (Trim 1-Pl). For each LCM sample, the cumulative number of '1s' for DMRs hypermethylated in first trimester placental tissues are on the x-axis, and the cumulative number of '1s' for DMRs hypermethylated in term placental tissues are on the y-axis (Methods). No relationship was found with gestational age in spiral arteries or endometrial cells for identified DMRs in placental tissues. Endo = endometrial cells, Sp = spiral arteries.

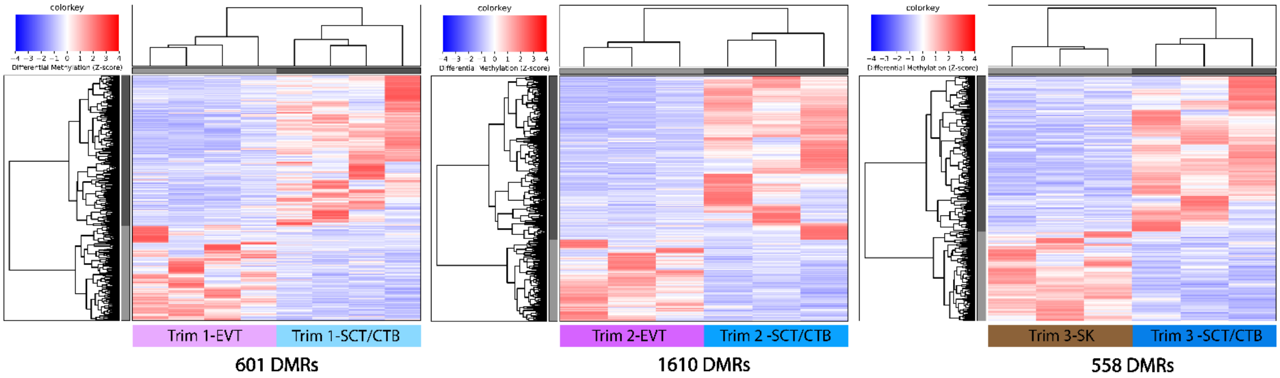

Supplementary Figure S3: DMRs identified between trophoblast populations collected at a comparable gestational age. Heatmaps visualizing unsupervised hierarchical clustering for autosomal DMRs with a fold change ≥2 between **1)** first trimester extravillous trophoblasts (EVTs) and syncytiotrophoblasts/cytotrophoblasts (SCTs/CTBs) (n=601) (left); **2)** second trimester EVTs and SCTs/CTBs (n=1610) (middle); and **3)** Term Syncytial knotting (SK) and SCTs/CTBs (n=558) (right).

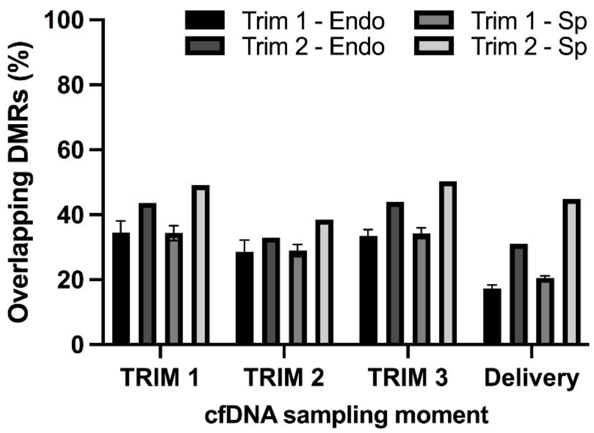

Supplementary Figure S4: Overlap of DNA methylation of endometrial cells and spiral arteries with DMRs identified in maternal cfDNA. The proportion of DMRs identified in maternal cfDNA in the different trimester and at delivery as compared to cfDNA from non-pregnant women, that overlap with DNA methylation in endometrial cells and spiral arteries based on our cumulative methylation score (Methods). The means and SDs are depicted. Overlap is considerably lower as compared to overlap with trophoblast cells (Figure 5C) and no increase is observed related to gestational age. Endo = endometrial cells, Sp = spiral arteries.

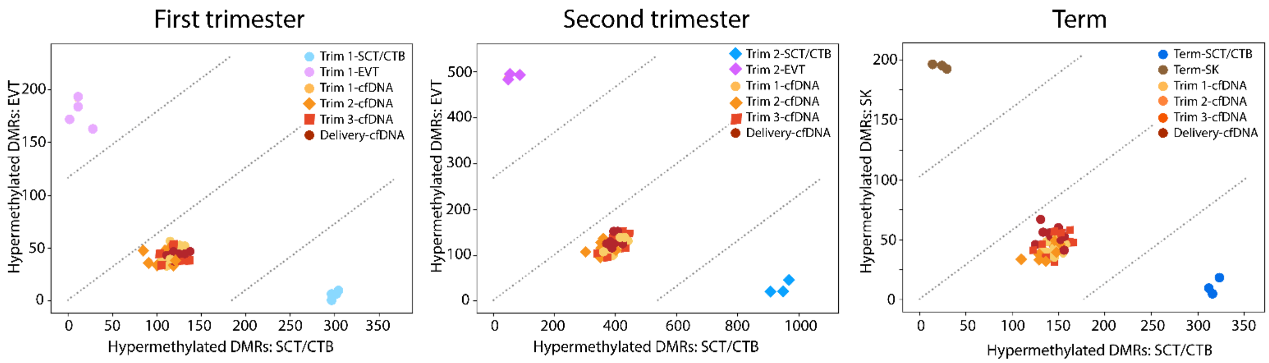

Supplementary Figure S5: DNA methylation in maternal cfDNA associated with specific trophoblast populations. Cumulative methylation scores for maternal cfDNA samples based on methylation of DMRs identified between different trophoblast populations with a comparable gestational age. We calculated a binary score for each identified DMR related to trophoblast population (Methods). In maternal cfDNA, we do not specifically distinguish DNA methylation markers from first (left) or second (middle) trimester extravillous trophoblasts (EVTs) as compared to first (left) or second (middle) trimester syncytiotrophoblasts/cytotrophoblasts (SCTs/CTBs), or from term (right) Syncytial knotting (SK) as compared to term SCTs/CTBs. Each dot represents one sample.

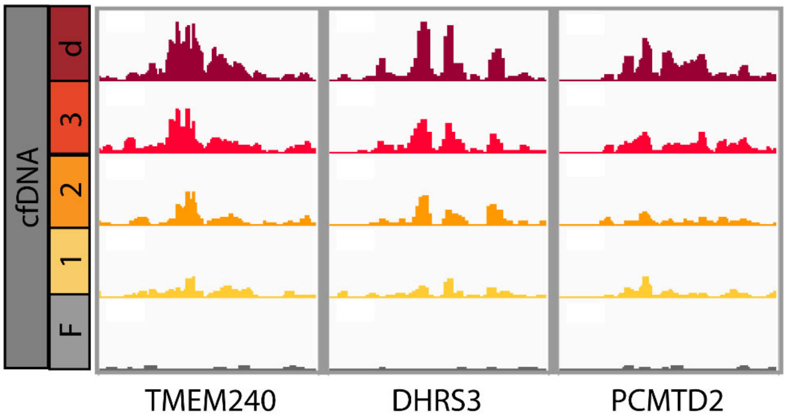

Supplementary Figure S6: Gene-tracks for three top-ranked placental-specific DMRs that were previously identified in cfDNA. F = non-pregnant women, 1 = first trimester, 2 = second trimester, 3 = third trimester, d = delivery.

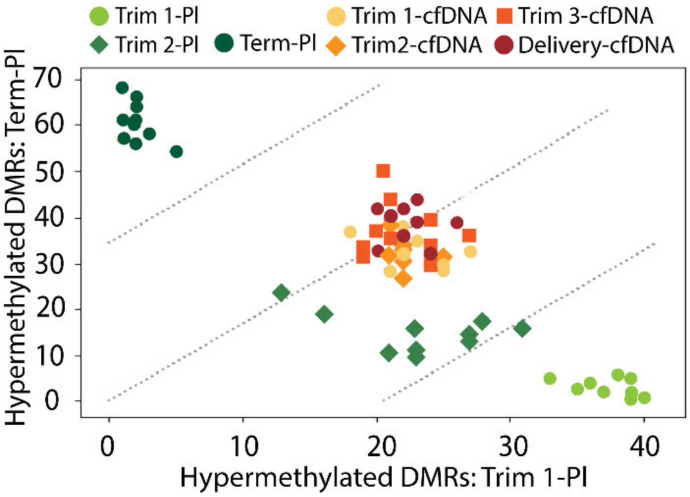

Supplementary Figure S7: DNA methylation in maternal cfDNA for all (n=110) DMRs identified between first trimester and term placental tissues. Overlap of DMRs identified between first trimester and term placental tissues was studied in maternal cfDNA collected in the different trimesters and at delivery. After generating a cumulative methylation score based on all identified placental DMRs, cfDNA samples from all trimesters show largely comparable overlap with DMRs hypermethylated in first trimester (x-axis) and term (y-axis) placental tissues. Each do represents one sample.
